# A saúde e a medicina portuguesas no primeiro quartel do século XX pelas lentes de Jorge Marçal da Silva

**DOI:** 10.1590/S0104-59702024000100056

**Published:** 2024-10-14

**Authors:** Manuel Mendes Silva

**Affiliations:** i Presidente, Secção de História da Medicina/Sociedade de Geografia de Lisboa. Lisboa – Portugal. mmendessilva@sapo.pt

**Keywords:** Jorge Marçal da Silva (1878-1929, Fotógrafo amador, Saúde e medicina, Sociedade portuguesa, século XX, Jorge Marçal da Silva (1878-1929, Amateur photographer, Health and medicine, Portuguese society, twentieth century

## Abstract

Este trabalho pretende ilustrar o panorama da saúde e da medicina em Portugal no primeiro quartel do século XX a partir do espólio fotográfico de Jorge Marçal da Silva, médico e fotógrafo amador, que tem sido preservado pela família. Da sua coleção, escolhemos uma seleção de fotografias que ilustram o *modus vivendi* da população portuguesa, no interior rural, no litoral piscatório e na vida citadina, bem como algumas das que retratam os ambientes hospitalares e a assistência médica na capital, no sentido de reflectir sobre a sua importância como fonte de reflexão historiográfica para a história da medicina portuguesa de Novecentos.

## Abstract (en)

This paper aims at illustrating the panorama of health and medicine in Portugal in the first quarter of the twentieth century using the photographic collection of Jorge Marçal da Silva, a doctor and amateur photographer, which has been preserved by his family. From his collection, we have chosen a selection of photographs that illustrate the modus vivendi of the Portuguese population, in the rural interior, on the fishing coast and in city life, as well as some that depict hospital environments and medical care in the capital, in order to reflect on their importance as a source for the historiographical reflection on the history of Portuguese medicine in the 1900s.

Jorge Marçal da Silva (1878-1929) nasceu em Lisboa, em 30 de junho de 1878, e morreu também em Lisboa, em 15 de maio de 1929, com 50 anos. Concluiu o curso de medicina em 1902, na Escola Médico-cirúrgica de Lisboa, com a tese *Feridas do coração, tratamento cirúrgico*, tendo sido aprovado com distinção de 15/20 valores. Foi colega de curso e amigo de Carolina Beatriz Ângelo (1878-1911), a primeira mulher a votar em Portugal, em 1910 (Serrano, 1903). Era casado e tinha quatro filhos.

Trabalhou no Hospital de São José, em Lisboa, como cirurgião substituto (1903-1906) e depois como cirurgião efetivo do Banco (1906-1910) (Mora, 11 jul. 2019). Assumiu as funções de facultativo assistente da seção cirúrgica, em 1910, e foi colocado no Hospital do Desterro durante cerca de um ano, passando depois para o Hospital de Dona Estefânia, onde foi responsável pelas enfermarias de mulheres, de onde veio a ser diretor. Marçal da Silva foi um dos médicos que transitaram entre vários espaços hospitalares administrados pelo Hospital de São José e Anexos/Hospitais Civis de Lisboa,^1^ o único hospital central público da capital, tendo desempenhado um papel de destaque nas patologias femininas. No Hospital de Dona Estefânia existiu, numa das salas, uma placa com o seu nome, em sua homenagem.

A sua atividade cirúrgica encontra-se documentada na publicação do Hospital Central, em particular, no *Boletim do Hospital de São José e Annexos* (1903-1912) e posteriormente no *Boletim dos Hospitais Civis de Lisboa* (1913-1923).

Esteve ao serviço de Portugal no quadro da Grande Guerra tendo sido cirurgião do Exército no Hospital Militar da Estrela de 1916 a 1919, onde chegou a ser capitão médico miliciano. Esteve também ligado ao ensino da enfermagem, tendo sido professor de anatomia e fisiologia na Escola Profissional de Enfermeiros de 1920 a 1928, no Hospital de São Lázaro, tornando-se seu director, entre 1924 e 1928.

Dedicava-se ainda à clínica privada, maioritariamente gratuita, na zona onde vivia, em Arroios (Mendes Silva, 2016a; Castro, Gens, 2023).

Possuía grande cultura; para além da fotografia, era melómano, amador de guitarra, apreciador de ópera e assinante do Teatro de São Carlos, frequentando-o assiduamente. Era um homem bom, humanista, benemérito, sendo respeitado nos meios médico e cultural da época. Quando morreu, no seu funeral, que saiu da Igreja de São Domingos até ao cemitério do Alto de São João, viam-se muitas pessoas, da zona de Arroios e da avenida Almirante Reis, que o choravam, tendo muitos comerciantes encerrado os seus estabelecimentos. Dele disse Dom Thomaz de Mello Breyner, seu colega muito prestigiado nesse tempo e um dos seus muitos amigos: “Morreu o melhor de nós todos” (Mendes Silva, 2015).

## Jorge Marçal da Silva: o fotógrafo e a fotografia

A captação da imagem pela câmara fotográfica sob o olhar de vários fotógrafos assumiu destaque na caracterização da mundividência portuguesa e teve como pioneiros fotógrafos profissionais, José Relvas (1858-1929), Joshua Benoliel (1873-1932), Afonso Chaves (1857-1926), Vicente (Madeira) (1827-1906), Alexandre Ramires, António Sena (1926-2001) e, mais recentemente, Artur Pastor (1922-1999), Luís Pavão, e Carmen Almeida, tema que tem sido abordado na historiografia da fotografia por autores como Augusto Silva Carvalho (1940), Fátima Nunes (2005) ou Hugo Pereira (2023), entre outros. São universos distintos de enquadramento da história da fotografia na representação de Portugal no seu espaço histórico dos séculos XIX e XX, mas ambos refletem a subjetividade subjacente a uma representação da câmara fotográfica, supostamente objetiva. Cada imagem é captada por uma câmara, que não se restringe apenas às suas lentes, mas também às lentes do seu operador, o fotógrafo.

No contexto da história da medicina, também a fotografia desempenhou este papel dual entre a realidade e a representação do visível, mas também entre o fotógrafo e o objeto fotografado.

A fotografia médica surgiu no contexto da psiquiatria e da dermatologia na segunda metade do século XIX (Neuse et al., 1996). Em 1852, o neurofisiologista francês Guillaume Duchenne (1806-1875), conhecido como Duchenne de Boulogne, publicou a obra *Le mécanisme de la physionomie humaine*, considerada como uma das primeiras publicações científicas, ilustrada com fotografias dos seus doentes e das experiências que realizava (Peres, Jardim, 2014). Em 1862, foi editada em Paris a obra de Alfred Hardy (1811-1893), *Clinique photographique de l’Hopital de Saint Louis* (Hardy, Montmeja, 1868). Em 1893, Albert Londe (1858-1917), do Hospital Salpêtrière, em Paris, publicou o primeiro tratado de fotografia médica “La photographie medicale: aplication aux sciences médicales et physiologiques” (Barata, 2016).

Em Portugal, Carlos May Figueira (1829-1913) foi um dos primeiros médicos a utilizar a microscopia nos anos 1860 (Barata, 2016) e a interessar-se também pela fotografia médica, por influência de Charles Robin (1821-1885), no laboratório de microscopia no qual estagiou, em 1856 (Costa, 1940, 1941). Tornou-se uma das figuras de referência no contexto português, no âmbito da microfotografia médica, não só no ensino, como também na investigação clínica. A sua primeira fotografia terá sido a de um doente que faleceu de febre amarela, aquando da epidemia que assolou Lisboa, entre 1856 e 1857, a que se seguiram outras para estudo da morfologia do fígado no decurso da evolução da doença, ou, ainda, outras ilustrativas de casos clínicos menos conhecidos na literatura médica (Figueira, 1864), nomeadamente, o hermafroditismo, alvo de investigação de António Cascais (2017). A valorização da cultura visual pela medicina por meio da fotografia foi alcançando cada vez mais expressão na ilustração dos artigos na imprensa médica e na bibliografia médica, na alvorada do século XX (Clode, 2009).

Marçal da Silva faz parte dessa geração de médicos que cultivaram, como amadores, o interesse pela fotografia, mas as suas lentes captavam uma mundividência mais abrangente da sociedade portuguesa de novecentos. Suas fotografias captavam o olhar das pessoas, os ambientes, os ofícios e a assistência aos doentes, nos hospitais, mas as suas lentes de médico não dissociavam a realidade captada pela câmara daquela que provavelmente seria a sua intenção, a de melhor entender como integrar a relação entre conhecimento e prática no exercício da sua profissão. Jorge Marçal da Silva foi um dos pioneiros da visão integrada de pessoas, objetos, profissionais e instituições, a que se seguiram outros, disso são exemplos, João Taborda (2013) e José Barata (2016).

**Figura 1:**
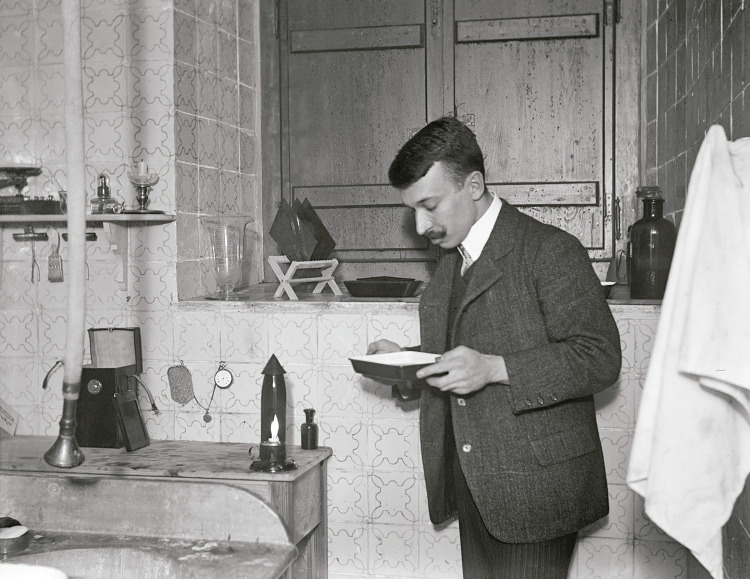
: Jorge Marçal da Silva no seu laboratório (Mendes Silva, 2016a, p.38)

Marçal da Silva desde cedo se dedicou à fotografia como autodidata. Revelava e preparava ele próprio as suas imagens no laboratório que tinha em sua casa (Figura 1). Lá registava metódica e minuciosamente todos os pormenores de data, local, características técnicas e outros, em pequenos livros que preenchia e guardava ciosamente (Mendes Silva, 2016a). Adquiria livros e assinava revistas sobre fotografia, mantinha-se atualizado sobre as técnicas e o seu alcance, para a medicina e para a relação entre a saúde, a doença, a pobreza, a higiene e a saúde pública. Era um cientista, além de artista.

Curioso e interessado como era, viajou bastante para a época, pois tinha meios para tal, tirou fotografias com a técnica que aprimorou ao longo do tempo em muitos locais de Portugal e alguns da Espanha raiana (Galiza e Andaluzia), sobre paisagens, costumes, feiras, ofícios, figuras, rostos, além de retratos da cidade onde vivia, Lisboa, da quinta onde morava, da família e dos hospitais onde trabalhou, bem como de alguns casos clínicos. As suas imagens, de lugares e temas tão variados, são um património histórico, sociológico, etnográfico, cultural, um testemunho de Portugal do início do século XX, nas suas diversas realidades e contrastes (Mendes Silva, 2013).

Infelizmente, as suas máquinas fotográficas desapareceram, mas permaneceram as quase duas mil fotografias, negativos e positivos em vidro, a maioria estereoscópicas, duas dezenas em cores.

Existem algumas produções monoscópicas e anáglifos, mas a técnica mais utilizada era, pouco frequente ao tempo, a esteroscópica, com imagens captadas em suporte de gelatina e prata, em vidro. Entre as duas imagens nos positivos em vidro está a sua numeração e caracterização de local e data, juntamente com a assinatura do autor, o que permite identificação pormenorizada (Castro, Gens, 2023).

Permanecem na coleção o *taxiphote*, equipamento para visualização binocular em relevo para observação dos positivos estereoscópicos em vidro, e o ampliador/projetor onde observava e mostrava as fotografias que realizava. Ficaram também os livrinhos com todos os seus registos e notas, como atrás foi referido, com a titulação das fotografias, locais, datas, horários, condições de luminosidade e características técnicas e eventuais outros pormenores de interesse, uma fonte de grande valor para a história da fotografia em Portugal (Castro, Gens, 2023).

O espólio fotográfico de Jorge Marçal da Silva foi doado ao Arquivo Fotográfico da Câmara Municipal de Lisboa, e parte dele foi integrado para a realização de uma exposição pela instituição, que explorou a dimensão imersiva de algumas das suas fotografias. Essa exposição esteve aberta ao público entre 17 de abril e 30 de setembro de 2023, tendo sido publicado o seu catálogo (Castro, Gens, 2023; Silva, s.d.).

As fotografias do Portugal rural, do interior, agrícola e protoindustrial, e do Portugal litoral, piscatório, ilustram como se vivia nessas regiões do país, bem como as implicações que essa forma de vida – com deficientes condições sanitárias – tinha na saúde das populações, além da falta de meios para prevenir, diagnosticar e tratar as doenças e os doentes.

**Figura 2:**
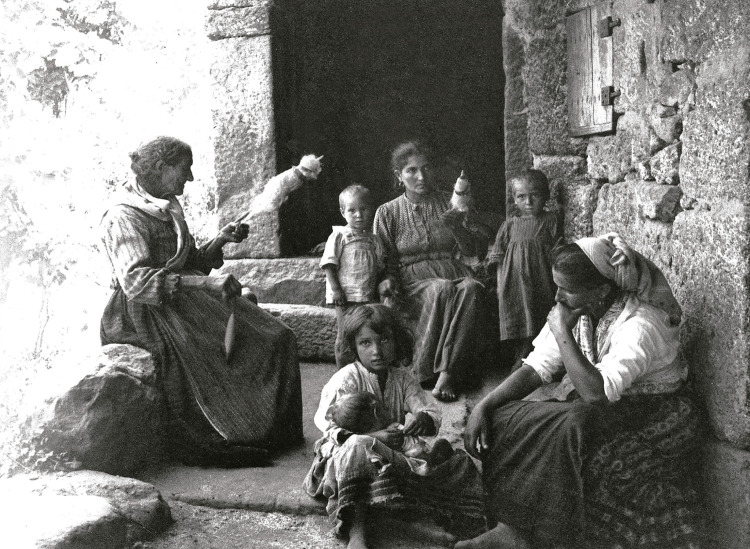
: Mulheres fiando, Taipas, 1919 (Mendes Silva, 2013, p.95)

**Figura 3:**
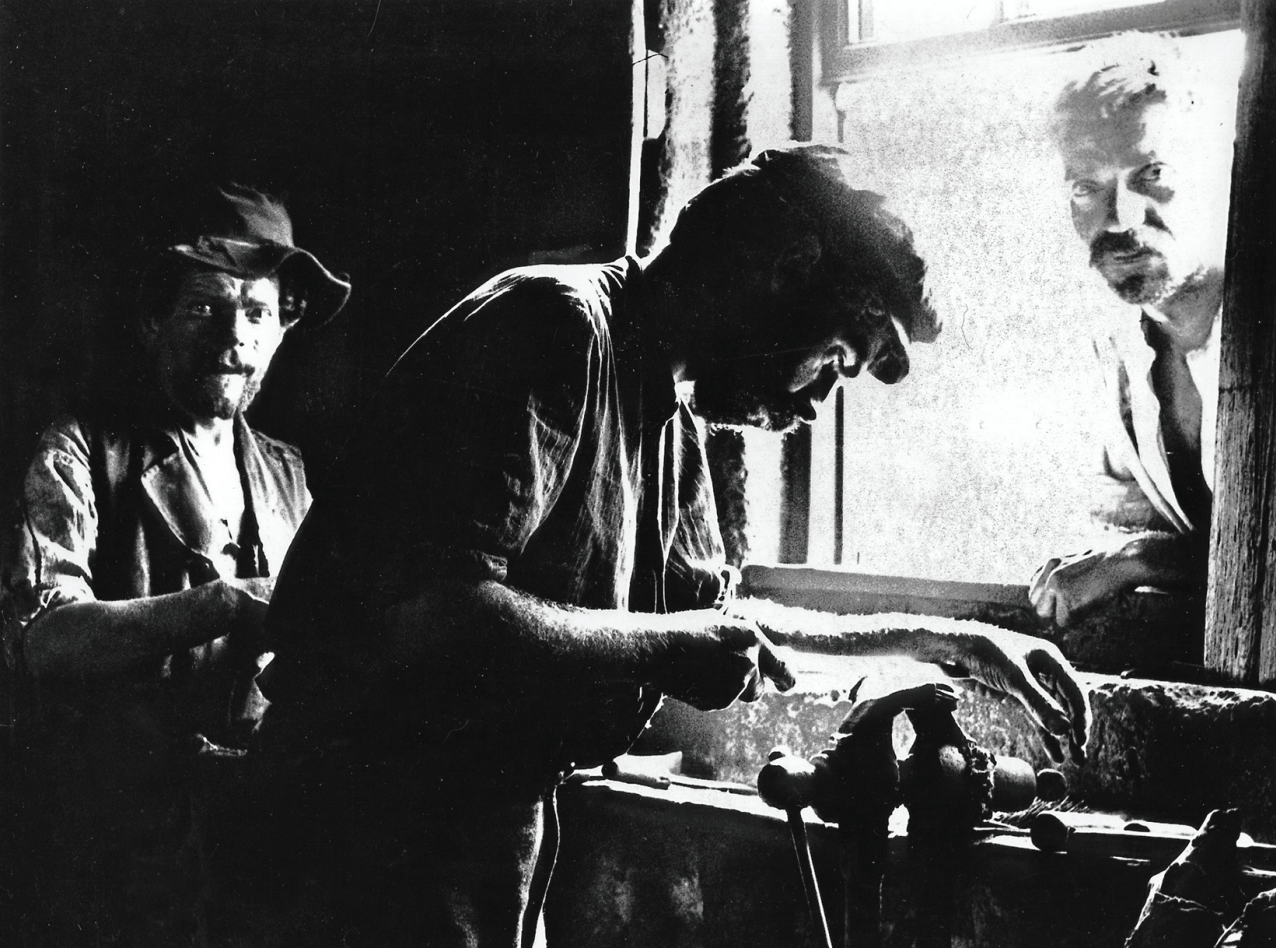
: Garfeiros, Taipas, 1907 (Mendes Silva, 2013, p.106)

Foram muito retratados por Marçal da Silva a agricultura, a pesca, a habitação, os ofícios, as feiras, as paisagens, os ambientes e os costumes da época (Mendes Silva, 2016b), o que denota um genuíno interesse de Marçal da Silva em registar a realidade social desse tempo e as suas implicações, incluindo na saúde. As Figuras 2, 3 e 4 são alguns exemplos.

**Figura 4:**
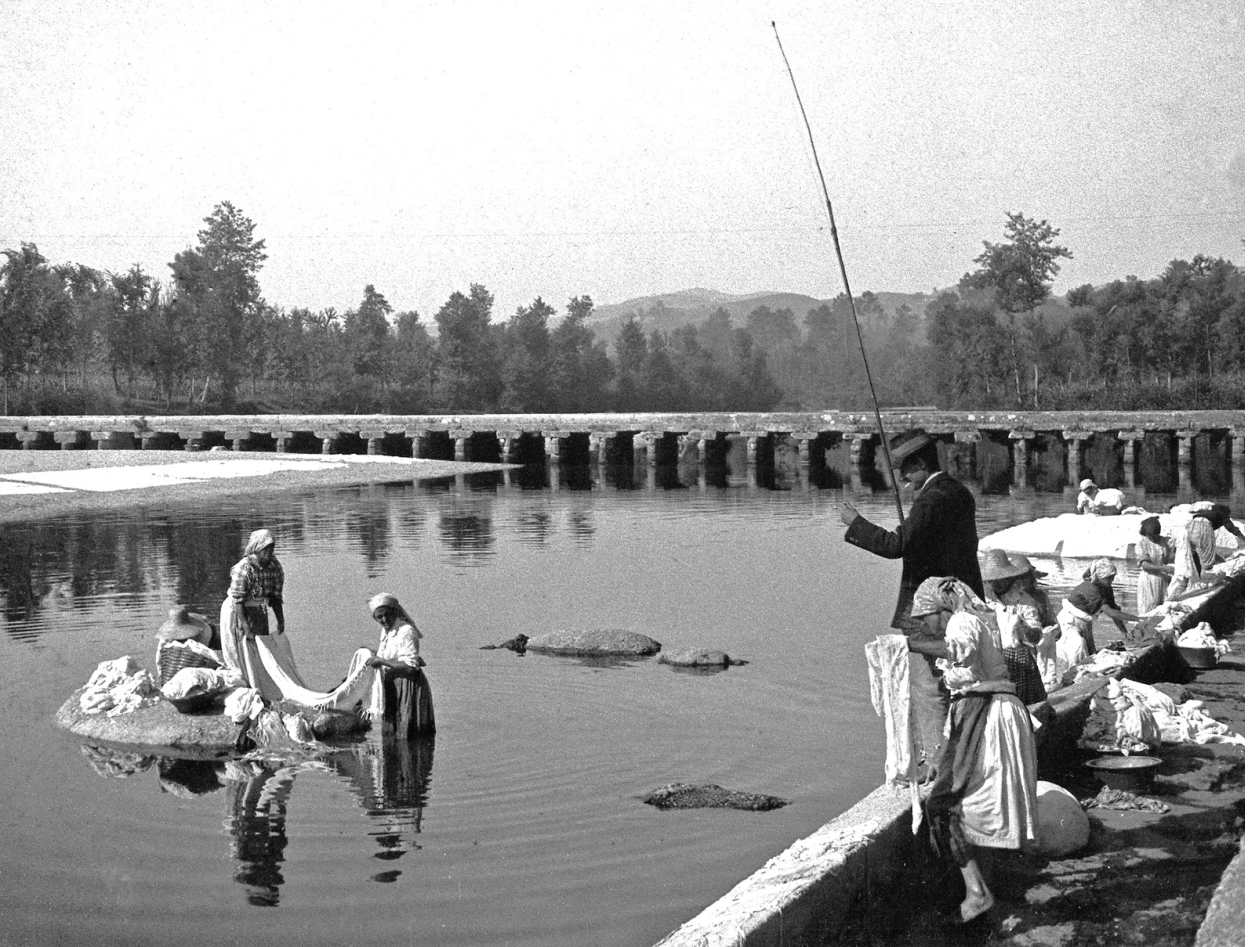
: Lavadeiras, Rio Ave, Taipas, 1919 (Mendes Silva, 2013, p.92)

**Figura 5:**
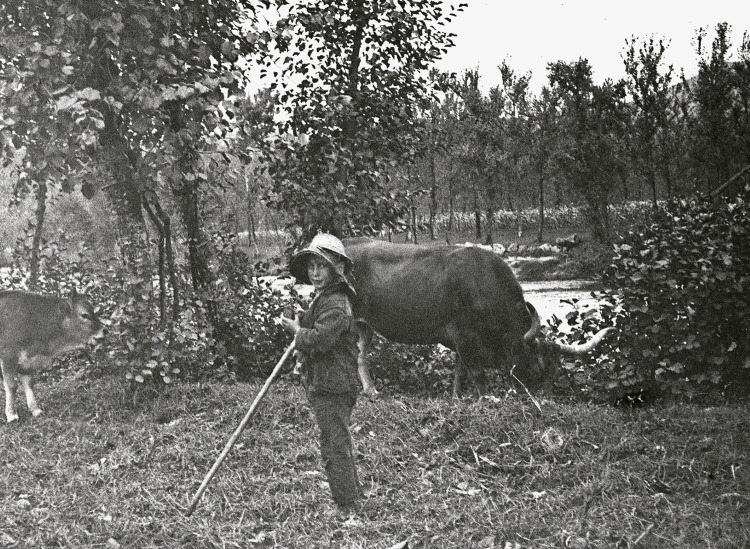
: Criança guardando gado, Taipas, 1923 (Mendes Silva, 2013, p.129)

Há cem anos, a vida era dura, o desenvolvimento escasso ou inexistente, a saúde precária. Sofriam-se as consequências da instabilidade política, da Grande Guerra, da crise económico-financeira, da epidemia da gripe espanhola. O trabalho infantil era a regra, com má nutrição, deficientes cuidados higiénicos, pouca cobertura médica, grande prevalência de doenças infectocontagiosas – incluindo a tuberculose – e alta taxa de mortalidade pediátrica e materno-infantil (Figura 5).

As malformações ficavam para a vida. Era frequente, também, a patologia traumática – quedas, agressões, coices, mordeduras, picadas, queimaduras –, bem como as neoplasias, que, se malignas, ainda que tratadas, eram quase sempre fatais. O alcoolismo e o tabagismo eram frequentes nos homens. A mortalidade e morbilidade por acidentes vasculares eram elevadas na população mais idosa.

**Figura 6:**
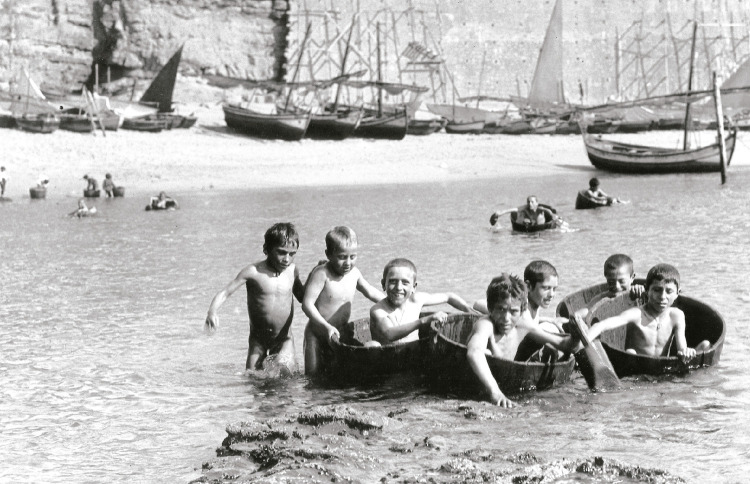
: Rapazes em celhas, Ericeira, 1908 (Mendes Silva, 2013, p.132)

A dureza da vida não significava, contudo, que não se realizassem festas populares, procissões e romarias, e que as crianças não tivessem imaginação para brincadeiras infantis (Figura 6).

Nas cidades do interior iam funcionando, com escassos meios, os hospitais das misericórdias. Mas eram os grandes hospitais de Lisboa, Porto e Coimbra que melhor funcionavam, dentro dos condicionalismos da época, e que acabavam por ser o terminal de drenagem assistencial para os doentes mais graves. Isso, além de algumas clínicas privadas para uns poucos privilegiados. Também era nessas cidades que funcionavam as escolas médico-cirúrgicas, em Lisboa e no Porto (até 1911), e a Faculdade de Medicina, na Universidade de Coimbra.

No espólio de Marçal da Silva, existem algumas fotografias do Hospital de São José e do Hospital de Dona Estefânia, onde trabalhou.

O Banco do Hospital de São José, em Lisboa, era um serviço de urgência médico-cirúrgica de referência no Sul do país e em nível nacional. O cirurgião-chefe, papel por ele assumido durante uns anos (Cirurgiões…, 1910), era muito respeitado pelos seus pares e pelo público, havendo fotografias do seu gabinete e do seu quarto, com uma banheira anexa – onde repousava e se banhava em alguns intervalos nas 24 horas de serviço.

**Figura 7:**
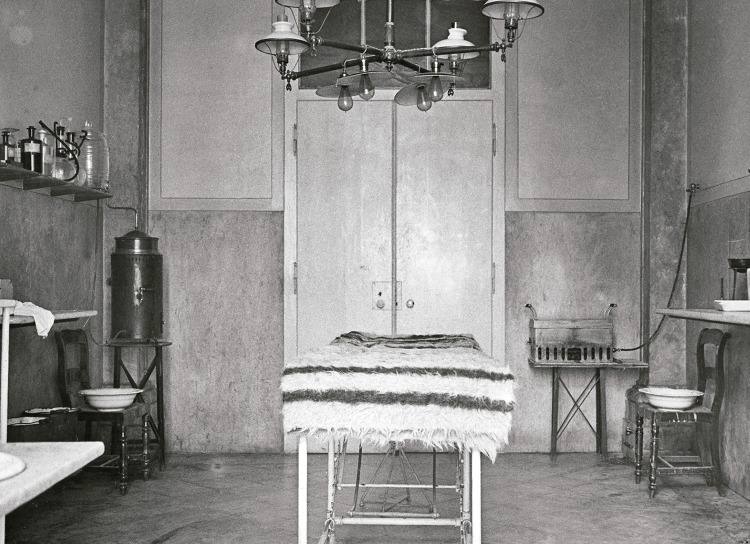
: Sala de operações do Banco, Hospital de São José, Lisboa, 1911 (Mendes Silva, 2013, p.34)

A sala de operações do Banco é retratada de duas formas complementares que ilustram as condições em que doentes e médicos coabitavam no espaço cirúrgico. Na Figura 7, ao centro, é possível ver na sala vazia a rudimentar marquesa de operações encimada por uma coberta, a qual serviria para tapar e aquecer os doentes, nomeadamente os que se encontravam em choque (Figura 7). Essa coberta, ou uma semelhante, pode ser também visualizada numa fotografia, de outro autor, retratando Miguel Bombarda (1851-1910) aquando da sua entrada no Banco, após ter sido baleado nas vésperas da Revolução Republicana, a 3 de outubro de 1910, um incidente que conduziu à sua morte.

A iluminação da sala faz-se por meio de um candeeiro central electrificado com oito lâmpadas, quatro mais quatro, que teria sido primitivamente a gás – ainda não havia *pantoffs*. Junto às paredes, forradas com algum tipo de oleado, estão um lavatório, bacias, prateleiras com material ou medicamentos, aparelhos primitivos de anestesia. O chão parece também forrado com oleado lavável. Ao fundo, a porta de madeira, pintada de branco, é uma porta comum, o que justifica a denominação de “sala de operações”, e não de “bloco operatório”.

**Figura 8:**
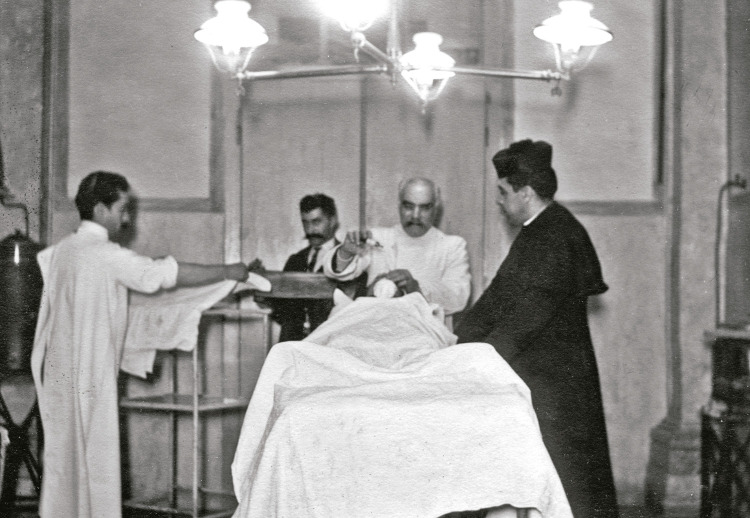
: Uma cirurgia na sala de operações do Banco, Hospital de São José, Lisboa, 1911 (Mendes Silva, 2016a, p.49)

A fotografia do início de uma cirurgia (Figura 8), em que o doente está a ser anestesiado e um médico (Senna Pereira) a pôr a mesa operatória, tem a presença do padre-cura, Luiz Figueiredo, e de um colega não identificado, atrás. Por que razão estaria o padre presente? Seria uma situação de grande gravidade que merecesse assistência eclesiástica? Seria a pedido do doente? Seria apenas uma visita do padre ao Banco?

Os trajes usados na sala de operações merecem realce: bata simples, traje de trabalho, ou de passeio, no caso do colega atrás; além do barrete do padre, não há máscaras, barretes cirúrgicos ou luvas. Não se vê nenhum(a) enfermeiro(a). A cirurgia estava entregue ao cuidado médico; o ensino e o treino especializado dos enfermeiros estavam em embrião.

**Figura 9:**
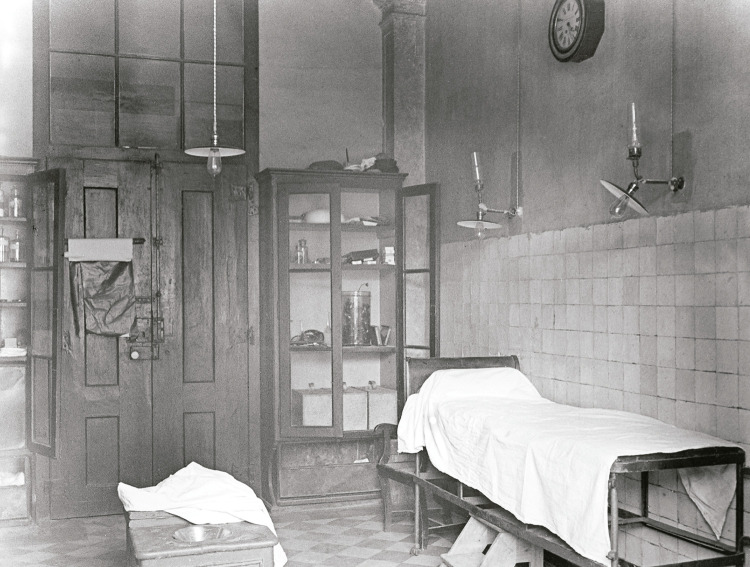
: Sala de curativos do Banco, Hospital de São José, Lisboa, 1911 (Mendes Silva, 2013, p.33)

A sala de curativos (Figura 9) tinha ao centro um banco e, junto à parede, uma marquesa. A iluminação era já elétrica, mas os candeeiros de parede tinham sido a gás. A porta da sala é de madeira, normal, e as paredes são revestidas de azulejos, havendo estantes e baús para guardar material. Um relógio de parede, se não estivesse avariado, indicava o tempo. Pendurado na porta, está o que parece ser um improvisado saco para sujos. Em cima de uma prateleira de trabalho, onde havia um fervedor, estão dois recipientes de água em vidro. O chão é de ladrilho. Não se vê nenhum lavatório. Ali se faziam as pequenas cirurgias, com os doentes sentados ou deitados, conforme as circunstâncias.

**Figura 10:**
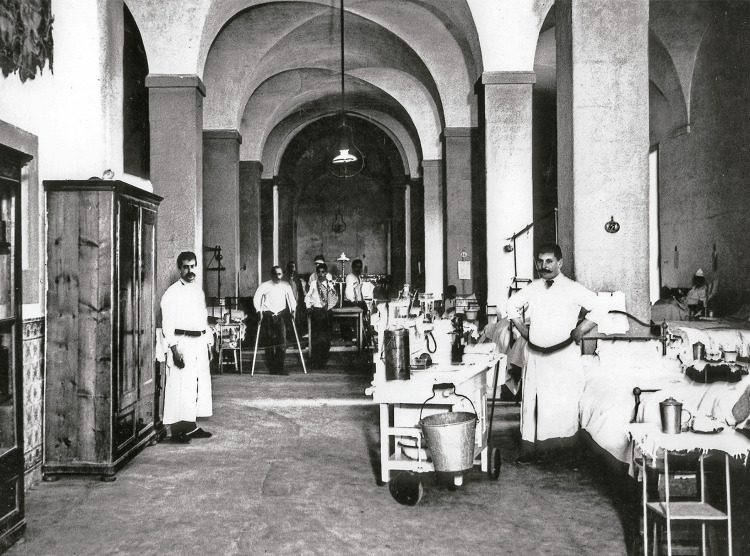
: Enfermaria, Hospital de São José, Lisboa, 1911 (Mendes Silva, 2013, p.35)

As enfermarias, em grandes salas conventuais de grande pé-direito, com numerosas camas em ferro e banquinhas de cabeceira ao lado, e por vezes uma cadeira em latão, eram geridas pelos enfermeiros que, conforme indicações médicas nas visitas periódicas, administravam os tratamentos e faziam os pensos, levando o carro, de cama em cama. A Figura 10 assim o mostra, mas, 60 anos depois, muito desse cenário ainda existia…

Jorge Marçal da Silva pouco retratou casos clínicos, ao contrário dos médicos amadores da sua geração, e quando o fez foi para apresentar situações invulgares ou resultados de cirurgias realizadas, como um caso de tumor da face antes e depois da cirurgia.

Esses são, pois, alguns poucos exemplos – que sensibilizam particularmente – do espólio que nos deixou esse médico, fotógrafo amador, que retratam os finais do século XIX e as primeiras décadas do século XX: as condições económicas, a organização social e o seu reflexo no campo da saúde, nomeadamente na explicação da prevalência de algumas doenças e da assistência aos doentes.

## Considerações finais

A fotografia de Jorge Marçal da Silva emerge como uma janela singular para a compreensão da sociedade portuguesa durante a primeira metade do século XX. Através de suas lentes, somos transportados para um panorama sanitário marcado por profundas assimetrias sociais, em que a pobreza e as condições precárias de higiene se entrelaçam. É um testemunho visual das realidades mundanas, mas também dos médicos da capital que, por meio da imagem fotográfica, procuravam prescrutar o quão essa representação visual ampliava os horizontes dos seus conhecimentos médicos e lhes permitia, na prática, inovar num consultório, numa enfermaria ou num Banco do hospital. Ao examinarmos de perto essas imagens, somos confrontados com a prevalência de certas doenças e do significado da assistência médica nos hospitais portugueses dos anos 1900. Ao apresentar a visão de Jorge Marçal da Silva, a forma como captava o mundo com as suas lentes, a sua câmara, a sua técnica e a sua curiosidade de médico e de fotógrafo amador, nesse período histórico, essas fotografias não são apenas documentos visuais, mas também ferramentas essenciais para uma análise e compreensão mais próxima da história da medicina e da sociedade portuguesa nas primeiras décadas do século XX.

## NOTA

1 Assim designado após 1913, na sequência da instauração do regime republicano, em Portugal.
